# Chromosome-Level Genome Assembly for *Acer pseudosieboldianum* and Highlights to Mechanisms for Leaf Color and Shape Change

**DOI:** 10.3389/fpls.2022.850054

**Published:** 2022-03-03

**Authors:** Xiang Li, Kewei Cai, Zhiming Han, Shikai Zhang, Anran Sun, Ying Xie, Rui Han, Ruixue Guo, Mulualem Tigabu, Ronald Sederoff, Xiaona Pei, Chunli Zhao, Xiyang Zhao

**Affiliations:** ^1^College of Forestry and Grassland, Jilin Agricultural University, Changchun, China; ^2^State Key Laboratory of Tree Genetics and Breeding, School of Forestry, Northeast Forestry University, Harbin, China; ^3^Southern Swedish Forest Research Centre, Faculty of Forest Science, Swedish University of Agricultural Sciences, Lomma, Sweden; ^4^Forest Biotechnology Group, Department of Forestry and Environmental Resources, North Carolina State University, Raleigh, NC, United States

**Keywords:** *Acer pseudosieboldianum*, PacBio SMRT, HiFi, genome assembly, Hi-C, TCPs

## Abstract

*Acer pseudosieboldianum* (Pax) Komarov is an ornamental plant with prominent potential and is naturally distributed in Northeast China. Here, we obtained a chromosome-scale genome assembly of *A. pseudosieboldianum* combining HiFi and Hi-C data, and the final assembled genome size was 690.24 Mb and consisted of 287 contigs, with a contig N50 value of 5.7 Mb and a BUSCO complete gene percentage of 98.4%. Genome evolution analysis showed that an ancient duplication occurred in *A. pseudosieboldianum*. Phylogenetic analyses revealed that Aceraceae family could be incorporated into Sapindaceae, consistent with the present Angiosperm Phylogeny Group system. We further construct a gene-to-metabolite correlation network and identified key genes and metabolites that might be involved in anthocyanin biosynthesis pathways during leaf color change. Additionally, we identified crucial teosinte branched1, cycloidea, and proliferating cell factors (TCP) transcription factors that might be involved in leaf morphology regulation of *A. pseudosieboldianum*, *Acer yangbiense* and *Acer truncatum*. Overall, this reference genome is a valuable resource for evolutionary history studies of *A. pseudosieboldianum* and lays a fundamental foundation for its molecular breeding.

## Introduction

Maple is a deciduous perennial tree or shrub belonging to the genus *Acer* of the Aceraceae family and is naturally distributed in Asia, Europe, North America, and the northern edge of Africa (van Gelderen et al., 1994; Yang et al., 2019; Grossman, 2021). In particular, the core distribution area of maple species has been in China, possessing abundant germplasm resources with more than 150 *Acer* L. species and accounting for more than half of the world’s maple resources, which are of great significance for further study of the evolutionary history of *Acer* species (Zhu and Lin, 2014; Ma et al., 2020). In the past few years, these *Acer* species have been widely introduced, domesticated and utilized because of their strong adaptability, resistance, and high ornamental and economic value (Rong and Xu, 2020; Areces-Berazain et al., 2021; Li et al., 2021b). Maple is not only used for wood and the pharmaceutical industry but also a well-known modern landscaping tree species with a large tree shape, beautiful leaf shape, gorgeous leaf color, peculiar fruit shape and abundant genetic variation, occupying an important role in urban ecosystems and landscape construction (Dong et al., 2019; Quambusch et al., 2021). Previous studies have shown that the root, leaf, bark, fruit and seed extracts of *Acer* species are rich in nutrient components, such as amino acids, fatty acids, mineral elements, and some key physiologically active substances, such as triterpene, chlorogenic acid and nervonic acids (He et al., 2021). In addition to medicinal value, many *Acer* species display varying leaf color types in the growth and development process, such as from green to yellow (such as *Acer catalpifolium* Rehde. and *Acer truncatum* Bunge) and green to red [*Acer mandshuricum* Maxim. and *Acer pseudosieboldianum* (Pax) Komarov]. In addition, there is abundant phenotypic variation in leaf shape in *Acer* species, and simple leaves with trilobation, quinquepartite, and heptalobus can be found in *Acer* species, which lays the foundation for further genetic improvement of *Acer* species (Royer et al., 2009; Couturier et al., 2012; Zhu et al., 2015). However, the majority of *Acer* trees face the risk of extinction due to frequent human activities and pest effects. Presently, the majority of *Acer* species, such as *Acer campestre* L., *A. yangbiense* Y. S. Chen & Q. E. Yang, and *Acer miaotaiense* P. C. Tsoong, are listed as endangered species by the Red List of Threatened Species of International Union for Conservation of Nature and Natural Resources (IUCN), and these *Acer* species are also listed as national protected plants in China (Li et al., 2018; Wahlsteen, 2021).

**FIGURE 1:**
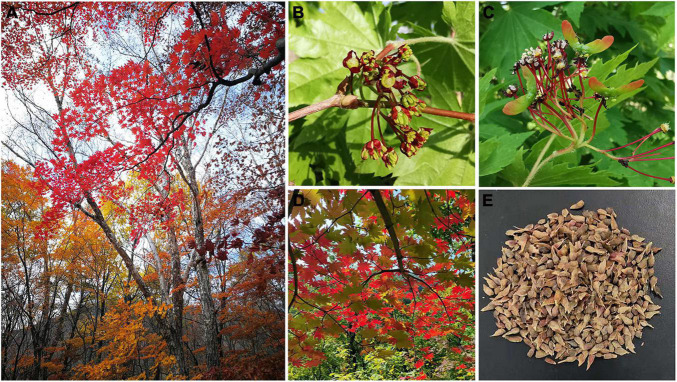
Photographs of *Acer pseudosieboldianum*. **(A)** Adult tree, **(B)** flower, **(C)** fruit, **(D)** leaf, and **(E)** seed.

*Acer pseudosieboldianum* (commonly known as Korean Maple), with chromosome numbers 2*n* = 2*x* = 26, is a precious deciduous tree and is only naturally distributed in Northeast China, the Russian Far East and the northern Korean Peninsula (Kim and Kim, 2016; Gao et al., 2021). As an important colored-leaf plant, it has been used for landscaping because of its beautiful samara and extremely red leaves in autumn (Figure 1); thus, it is considered one of the most promising native tree species for landscaping in Northeast China. Moreover, *A. pseudosieboldianum* is mainly distributed in cold temperate zones and can withstand temperatures lower than −30°C in winter in Northeast China. It is also endowed with unique adaptability and cold resistance compared with related *Acer* species (Sun et al., 2007). *A. pseudosieboldianum* might be a potential red-leaf maple resource for landscaping and future crossbreeding studies in the genus *Acer*. Currently, this species has been listed as an endangered species by the Chinese government and subjected to *ex situ* conservation, necessitating the importance of studying *A. pseudosieboldianum* for further development and utilization (Wang et al., 2014).

The ornamental value of *Acer* species is mainly determined by leaf color and leaf shape. Leaf color formation has been well characterized in many plants and is mainly regulated by three key pigments, chlorophyll, carotenoid and anthocyanin (Zhang Q. et al., 2020; Zhang Z. et al., 2020). Anthocyanins are widely distributed in leaves, flowers, fruits and roots in many plants and are considered the main sources of purple, blue and red formation (Jiao et al., 2020; Li et al., 2020). Their synthesis is mainly affected by R2R3-MYB, bHLH and WD40 transcription factors (TFs), and these TFs form an MYB-bHLH-WD40 (MBW) complex to regulate the key structural genes in anthocyanin synthesis pathways (Zhang and Hülskamp, 2019; Qin et al., 2020). Presently, the molecular mechanism of leaf color change has only been studied in some *Acer* species because of the lack of genome information. Additionally, the leaf shape of *A. pseudosieboldianum* varies from 9 to 11 fissures. The teosinte branched1, cycloidea, and proliferating cell factor (TCP) proteins, plant-specific bHLH TFs, play significant regulatory functions in plant morphogenesis, such as leaf development, collateral formation, flower development and seed germination (Sun, 2020; Zhao Y. F. et al., 2020; Zhao et al., 2021). Presently, the TCP family of genes has been found in many plants and demonstrated to participate in leaf development control, such as *Panicum virgatum* (Zheng et al., 2019), *Acer palmatum* (Zhu et al., 2021), and *Populus euphratica* (Ma et al., 2016). However, it remains unknown whether TCPs are also involved in leaf morphology regulation in *A. pseudosieboldianum*.

To date, *A. yangbiense* was the first species to undergo whole-genome sequencing among Aceraceae species, followed by *A. truncatum*, but there is still a lack of high-quality whole-genome sequences for this family, particularly for *A. pseudosieboldianum*. To accelerate the molecular breeding and usage of this precious species, in the present study, we assembled a chromosome-level genome of *A. pseudosieboldianum* (2*n* = 26, 690 Mb) combining Pacific Biosciences (PacBio) HiFi long reads. Using this high-quality genome, we conducted a comparative genomic analysis with the reported *A. yangbiense* and *A. truncatum* genomes. To better understand the molecular mechanism of leaf color formation, we also identified the key structural genes and metabolites that are involved in anthocyanin biosynthesis pathways, combining transcriptome and metabolome analyses. Moreover, we further identified the genes encoding the TCP family from the *A. pseudosieboldianum*, *A. yangbiense*, and *A. truncatum* genomes, which will contribute to further studies related to leaf shape regulation in the *Acer* species. The high-quality genome assembled herein serves as an important support for further functional gene digging in *A. pseudosieboldianum*. Also, this chromosome-level genome of *A. pseudosieboldianum* not only provides meaningful genetic information for *Acer* species evolution but also lays the foundation for molecular breeding studies of this precious species.

## Materials and Methods

### Plant Materials and Sequencing

For genome sequencing, fresh leaf materials were harvested from the best-growing *A. pseudosieboldianum* tree planted in the nursery of Hongshi Forestry Bureau, Huadian, China (Figure 1) and immediately frozen in liquid nitrogen, followed by preservation at −80 °C in the laboratory prior to DNA extraction. High-quality genomic DNA was extracted from leaves using a modified CTAB method (Porebski et al., 1997). RNase A was used to remove RNA contaminants. The quality and quantity of the extracted DNA were examined using a NanoDrop 2000 spectrophotometer (NanoDrop Technologies, Wilmington, DE, United States) and electrophoresis on a 0.8% agarose gel, respectively. Single-molecule real-time (SMRT) long-read sequencing was performed at Frasergen Bioinformatics (Wuhan, China) with a PacBio sequencer (Pacific Biosciences, Menlo Park, CA, United States). The SMRT Bell library was prepared using the SMRTbell Express Template Prep Kit 2.0. In total, one (15–20 kb) SMRT Bell library was constructed. Genomic DNA (∼5 μg) was mechanically sheared to fragments of approximately 15–20 kb using a Covaris g-TUBE. The fragment size distribution was assessed using Femto Pulse. Size selection of SMRT Bell templates was performed using a BluePippin size-selection system (Sage Science, Beverly, MA, United States) to enrich for large fragments (>25 kb). The quality and quantity of the size-selected libraries were assessed on a Femto Pulse and a Qubit fluorometer (Life Technologies, Carlsbad, CA, United States), respectively. The library was sequenced using a PacBio Sequel II instrument on PacBio SMRT cells 8 M (Pacific Biosciences, acquiring one movie of 15 h per SMRT cell). In total, 20.97 Gb (30.37× of the genome) HIFI read sequences (average length: 12,235 bp) were generated. The Illumina library with insert sizes of 350 bp was arranged with a Genomic DNA Sample Preparation kit from Illumina. It was then sequenced using an Illumina platform and yielded 59.06 Gb (85.55× of the genome) of raw sequence data.

### RNA Isolation and Iso-Seq Sequencing

To obtain sufficient materials for each RNA sample, we pooled plant tissue (root, bark, leaf, and fruit) from the *A. pseudosieboldianum* tree. Samples were placed on dry ice during sample collection and stored at −80 °C until RNA isolation. Samples were ground in liquid nitrogen, and total RNA was extracted using TRIzol reagent (Invitrogen, Carlsbad, CA, United States) according to the manufacturer’s protocol. RQ1 DNase (Promega, Madision, WL, United States) was used to remove DNA. cDNA libraries were prepared using the Clontech SMARTer ^®^ cDNA synthesis kit according to the manufacturer’s recommendations. One microgram of total RNA was used for each of the tissue samples. The cDNA products were purified with AMPure PB beads, and quality control (QC) was performed on a BioAnalyzer 2100 (Agilent). One to five micrograms of purified amplicons were subjected to Iso-Seq SMRT Bell library preparation^1^. A total of 34.48 Gb was sequenced on the PacBio Sequel II platform with 30-h movies.

### Genome Feature Estimation From *k*-Mer Analysis

The short reads from the Illumina platform were quality-filtered by HTQC v1.92.310 (Yang et al., 2013) using the following method. First, the adaptors were removed from the sequencing reads. Second, read pairs were excluded if any one end had an average quality lower than 20. Third, the read ends were trimmed if the average quality was lower than 20 in the sliding window size of 5 bp. Finally, read pairs with any end shorter than 75 bp were removed. The quality-filtered reads were used for genome size estimation. We generated the 15-mer occurrence distribution of sequencing reads from short libraries using the *k*-mer method. Then, we estimated the genome size to be approximately 558.49 Mb, and the proportion of repeat sequences and heterozygosity rate of the genome were determined to be approximately 80.41 and 1.10%, respectively, using GCE (v.1.0.2) (Liu et al., 2013).

### Genome Assembly and Quality Evaluation

With one SMRT cell line on the PacBio Sequel II platform, we generated 20.97 Gb (30.37× of the genome) HiFi reads. All HiFi read data were used for the genome assembly of *A. pseudosieboldianum*. The HiFiasm (Cheng et al., 2021) software package (version 0.2.0) was used to assemble the *A. pseudosieboldianum* genome with default parameters. To correct errors in the primary assembly, we used Illumina-derived short reads to polish the genome by pilon (Walker et al., 2017) (v1.22), and 3D-DNA software (Dudchenko et al., 2017) was employed to refine the initial genome assembly using high-throughput chromosome conformation capture (Hi-C) data to remove heterozygosity. Finally, the *A. pseudosieboldianum* genome assembly had a total length of approximately 862.66 Mb, which accounted for ∼154.46% of the genome size estimated by *k*-mer analysis, containing 1,042 contigs with an N50 of 5.42 Mb.

For anchored contigs, 63.75 Gb of clean read pairs were generated from the Hi-C library and mapped to the polished *A. pseudosieboldianum* genome using BWA (bwa-0.7.17) (Li, 2013) with default parameters. Paired reads with mates mapped to a different contig were used to perform Hi-C-associated scaffolding. Self-ligation, non-ligation and other invalid reads, such as PCR amplification, random breaks, large, small and extreme fragments, were filtered. We then successfully clustered 1,042 contigs into 13 groups with the agglomerative hierarchical clustering method in Lachesis (Burton et al., 2013). Lachesis was further applied to order and orient the clustered contigs. We successfully ordered and oriented 249 contigs that were 690.24 Mb in length. Then, Purge Haplotigs (Roach et al., 2018) was used to filter redundant sequences due to heterozygosity. Finally, we obtained the first chromosomal-level high-quality assembly, with chromosomal lengths from 38.69 to 74.29 Mb containing 97.94% of the total sequence.

To examine the assembly integrity, the HiFi reads were again aligned onto the final assembly using minimap2 (v2.5) (Li, 2018) with default parameters. A total of 99.43% of raw reads could be mapped. The assembled genome was also subjected to BUSCO (v3.0.2) (Simão et al., 2015) with the gene sets of OrthoDB to evaluate the completeness of the genome. Overall, 98.4% complete and 0.6% partial BUSCOs were identified in the assembled genome. By aligning short reads from the Illumina platform to the genome, the high alignment ratio and single peak insertion length distribution demonstrated the high quality of the contig assembly.

### Annotation of Repetitive Sequences

The two methods were combined to identify the repeat contents in our genome, homology-based and *de novo* prediction. For the homology-based analysis, we identified the known TEs within the *A. pseudosieboldianum* genome using RepeatMasker (open-4.0.9) (Tarailo-Graovac and Chen, 2009) with the Repbase TE library (Jurka, 2000). RepeatProteinMask (Tarailo-Graovac and Chen, 2009) searches were also conducted using the TE protein database as a query library. For *de novo* prediction, we constructed a *de novo* repeat library of the *A. pseudosieboldianum* genome using RepeatModeler (Abrusán et al., 2009), which can automatically execute two core *de novo* repeat-finding programs, namely, RECON (v1.08) (Bao and Eddy, 2002) and RepeatScout (v1.0.5) (Price et al., 2005), to comprehensively conduct, refine and classify consensus models of putative interspersed repeats for the *A. pseudosieboldianum* genome. Furthermore, we performed a *de novo* search for long terminal repeat (LTR) retrotransposons against the *A. pseudosieboldianum* genome sequences using LTR_FINDER (v1.0.7) (Zhao and Hao, 2007). We also identified tandem repeats using the Tandem Repeat Finder (TRF) package (Gary, 1999) and non-interspersed repeat sequences, including low-complexity repeats, satellites and simple repeats, using RepeatMasker (Tarailo-Graovac and Chen, 2009). Finally, we merged the library files of the two methods and used RepeatMasker to identify the repeat contents.

### Annotation of Protein Coding Genes

We predicted protein-coding genes in the *A. pseudosieboldianum* genome using three methods: *ab initio* gene prediction, homology-based gene prediction and RNA-Seq-aided gene prediction. Prior to gene prediction, the assembled *A. pseudosieboldianum* genome was hard and soft masked using RepeatMasker (Tarailo-Graovac and Chen, 2009). We adopted Augustus (v3.3.1) (Stanke et al., 2006) and Genescan (Burge and Karlin, 1997) to perform *ab initio* gene prediction. Models used for each gene predictor were trained from a set of high-quality proteins generated from the RNA-Seq dataset. We used Exonerate (v2.2.0) (Slater and Birney, 2005) to conduct homology-based gene prediction. First, the protein sequences were aligned to our genome assembly, and coding genes were predicted using Exonerate (Slater and Birney, 2005) with default parameters. To carry out RNA-Seq-aided gene prediction, we first assembled clean RNA-Seq reads into transcripts using TopHat (v2.1.1) (Trapnell et al., 2009), and the gene structure was formed using Cufflinks (v2.2.1) (Trapnell et al., 2010). Finally, Maker (v3.00) (Campbell et al., 2014) was used to integrate the prediction results of the three methods to predict gene models. The output included a set of consistent and non-overlapping sequence assemblies, which were used to describe the gene structures. In total, 29,679 protein-coding genes with an average length of 3,817.09 bp were predicted in the assembled *A. pseudosieboldianum* genome.

Gene functions were inferred according to the best match of the alignments to the National Center for Biotechnology Information (NCBI) Non-Redundant (NR), TrEMBL, InterPro and Swiss-Prot protein databases using BLASTP (NCBI blast v2.6.0+) (Camacho et al., 2009) and the Kyoto Encyclopedia of Genes and Genomes (KEGG) database with an *E*-value threshold of 1E-5. The protein domains were annotated using PfamScan (Mistry et al., 2007) and InterProScan (v5.35-74.0) (Jones et al., 2014) based on the InterPro protein databases. The motifs and domains within gene models were identified by Pfam databases (Punta et al., 2012). Gene Ontology (GO) IDs for each gene were obtained from Blast2GO (Conesa et al., 2008). In total, approximately 27,531 (approximately 92.76%) of the predicted protein-coding genes of *A. pseudosieboldianum* could be functionally annotated with known genes, conserved domains, and Gene Ontology terms.

### Annotation of Non-coding RNA Genes

We used tRNAscan-SE algorithms (Lowe and Eddy, 1997) with default parameters to identify the genes associated with tRNA, which is an adaptor molecule composed of RNA that is used in biology to bridge the three-letter genetic code in messenger RNA (mRNA) with the twenty-letter code of amino acids in proteins. For rRNA identification, we first downloaded rRNA sequences from closely related species from the Ensembl database. Then, rRNAs in the database were aligned against our genome using blastn (Camacho et al., 2009) with a cutoff *E*-value < 1e-5, identity ≥ 85% and match length ≥ 50 bp. snoRNAs are a class of small RNA molecules that guide chemical modifications of other RNAs, mainly ribosomal RNAs, transfer RNAs and small nuclear RNAs. MiRNAs and snRNAs were identified using Infernal (Nawrocki et al., 2009) software against the Pfam database (Punta et al., 2012) with default parameters.

### Comparative Genome Analysis

To cluster families from protein-coding genes, proteins from the longest transcripts of each gene from *A. pseudosieboldianum* and other closely related species, including *A. yangbiense*, *A. truncatum*, *Xanthoceras sorbifolium*, *Dimocarpus longan*, *Pistacia vera*, *Sclerocarya birrea*, *Prunus dulcis*, *Arabidopsis thaliana*, *Vitis vinifera*, *Citrus sinensis*, *Citrus unshiu*, *Citrus clementina*, *Prunus avium*, and *Oryza sativa*, were used. All proteins were extracted and aligned to each other using BLASTP (Camacho et al., 2009) programs (ncbi blast v2.7.1) with a maximal *E*-value of 1e-5. To exclude putative fragmented genes, we filtered out genes with an identity less than 30%, coverage less than 50% and those encoding protein sequences shorter than 100 amino acids. The OrthoMCL (v14-137) (Li et al., 2003) method was used to cluster genes from these different species into gene families with the parameter “-inflation 1.5.”. As a result, 16,229 gene families were constructed for *A. pseudosieboldianum* in this work, and 560 genes were identified as single-copy orthologous genes.

To reveal phylogenetic relationships among *A. pseudosieboldianum* and other closely related species, protein sequences from 560 single-copy orthologous genes were used for phylogenetic tree reconstruction. The protein sequences of the single-copy orthologous genes were aligned with the MUSCLE (v3.8.31) (Edgar, 2004) program, and the corresponding coding DNA sequence (CDS) alignments were generated and concatenated with the guidance of protein alignment. RAxML (v8.2.12) (Stamatakis, 2014) was used to construct the phylogenetic tree with the maximum likelihood method. The phylogenetic relationship of other closely related species was consistent with previous studies. Estimation of divergence times was obtained using the mcmctree program in PAML (v4.9e) (Yang, 2007) and r8s (Sanderson, 2003), with seven corrected divergence time point from the TimeTree website^2^ : *P. vera* vs. *S. birrea* (45–97 Mya), *C. unshiu* vs. *S. birrea* (62–84 Mya), *A. thaliana* vs. *X. sorbifolium* (96–104 Mya), *P. avium* vs. *A. thaliana* (98–117 Mya), *V. vinifera* vs. *A. thaliana* (107–135 Mya), *O. sativa* vs. *P. dulcis* (115–308 Mya), *C. sinensis* vs. *C. clementina* (1–6 Mya), and two corrected divergence time point from *A. truncatum* genome article (Ma et al., 2020): *A. truncatum* vs. *A. yangbiense* (4–17 Mya), *A. truncatum* vs. *Citrus sinensisv* (56–79 Mya).

According to the divergence times and phylogenetic relationships, 678 gene families were significantly expanded in *A. pseudosieboldianum* (*p* < 0.05). Those expanded gene families included 81 significantly enriched (*q*-value < 0.05) KEGG pathways. Based on the identified gene families and the constructed phylogenetic tree with the predicted divergence time of those species, we used CAFÉ (Bie et al., 2006) to analyze gene family expansion and contraction. In CAFÉ, a random birth and death model is proposed to study gene gain or loss in gene families across a specified phylogenetic tree. Then, a conditional *p*-value is calculated for each gene family, and families with a conditional *p*-value less than 0.05 are considered to have an accelerated rate for gene gain or loss. These expansion and contraction gene families in *A. pseudosieboldianum* (*p*-value ≤ 0.05) were mapped to KEGG pathways for functional enrichment analysis, which was conducted using the enrichment methods. This method implemented hypergeometric test algorithms, and the *q*-value (false discovery rate, FDR) was calculated to adjust the *p*-value using the R package^3^.

### Whole-Genome Duplication Event and Collinearity Analysis

Based on the genome and annotation documents of three *Acer* species, including *A. pseudosieboldianum*, *A. yangbiense* and *A. truncatum*, Mummer (v4.0.0beta2) (Delcher et al., 2003) was used for sequence alignment and sorting between the genomes of the three species. Then, Mcscan software^4^ (Tang et al., 2008) was further used to obtain collinear genes and collinear gene blocks. The whole-genome duplications (WGDs) of three species were identified by *K*_s_ (synonymous mutation rate) methods (Hurst, 2002).

### Transcription and Metabolite Analysis

Mature leaves at the green leaf (GL), half-red (HRL) leaf and red leaf (RL) stages were collected on June 15, September 25, and October 10, 2020, for transcriptomic and metabonomic analysis, respectively. In total, nine cDNA libraries were constructed and used for sequencing using the Illumina platform. The clean reads were obtained by removing adapter and low-quality reads from raw data and then further aligned with the *A. pseudosieboldianum* reference genome using Hisat2 (v2.1.0) (Kim et al., 2019) software. FeatureCounts (Liao et al., 2014) software was used for gene expression quantification to obtain the raw count matrix. Differential expression analysis was performed using DEseq2 (Love et al., 2014) software to obtain the differentially expressed genes (DEGs) in three comparisons, with cutoff values of | log_2_(fold change)| ≥ 1 and an adjusted *p*-value < 0.05. The gene function annotation and enrichment of DEGs were implemented with the common databases KEGG, NR, Swiss-Prot, Tremble, KOG, GO, and Pfam.

For the metabolomic analysis, collected fresh leaf samples (three biological replicates per stage) were immediately loaded into a precooled centrifuge tube and frozen with liquid nitrogen. Then, all the samples were crushed into a powder prior to subsequent ultra-performance liquid chromatography/tandem mass spectrometry (UPLC-MS/MS) (Garcia and Barbas, 2011) analysis. Analyst 1.6.3 software was used for qualitative and quantitative analysis of the mass spectrum data on the basis of the local metabolic database to obtain the original metabolite data. For quality control (QC) analysis, one quality control sample was inserted into each of the 10 tests and analysis samples to monitor the repeatability of the analytic process. Partial least-squares discriminant analysis (OPLS-DA) (Thévenot et al., 2015) was employed to screen variation components. The OPLS-DA model was further used to identify the differentially accumulated metabolites (DEMs) between the comparisons with the parameter of a variable importance in projection (VIP) value ≥ 1 and a fold change ≥ 2 or ≤0.5.

### Identification of Teosinte Branched1, Cycloidea, and Proliferating Cell Factors and Expression Analysis

We selected two *Acer* species (*A. yangbiense* and *A. truncatum*) to carry out a comparative TCP gene family analysis together with *A. pseudosieboldianum*. The publicly available *A. yangbiense* and *A. truncatum* whole-genome data were downloaded from the National Center for Biotechnology Information (NCBI) database^5^. Identification of the TCP gene family was performed by the two-step BLAST method. First, the sequences of 24 Arabidopsis *TCPs* obtained from the Arabidopsis Information Resource (TAIR)^6^ were used to search potential *A. pseudosieboldianum*, *A. yangbiense*, and *A. truncatum* TCPs with TBtools software (*e*-value, 1e-5) (Chen et al., 2020). All possible TCPs were further identified by the NCBI BLASTP process (*e*-value, 1e-5) (Camacho et al., 2009). The final candidate TCP proteins were further confirmed with the TCP domain (accession no. PF03634) in the Pfam database (Punta et al., 2012). Amino acid sequences of the candidate TCPs were used for multiple alignment and to construct the phylogenetic tree using MEGA (version 7.0) (Sudhir et al., 2016) software. Collinearity analysis for identified TCPs in three *Acer* species was performed using TBtools software (Chen et al., 2020).

The transcriptome data in different tissues of *A. yangbiense* and *A. truncatum* were obtained from the NCBI SRA with accession numbers PRJNA524417 and PRJNA557096, respectively. To analyze the expression of candidate TCPs in different tissues, Hisat2 (v2.0.12) (Kim et al., 2019) software was employed to separately align the RNA-seq data sets of different tissues to three genera of *Acer* species reference genomes. FeatureCounts software (Liao et al., 2014) was used to count the read numbers mapped to each gene. The standardized count matrix of RNA-Seq data was further used to display the expression level of TCPs in different tissues in three *Acer* species.

## Results

### Genome Sequencing and Assembly

**FIGURE 2:**
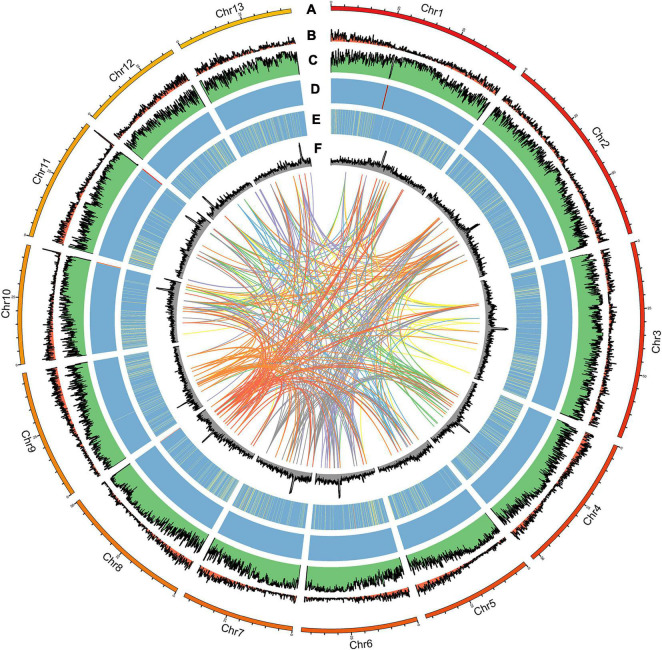
Distribution of *A. pseudosieboldianum* genomic features. **(A)** Circular representation of the chromosome, **(B)** gene density, **(C)** repeat density, **(D)** rRNA, **(E)** tRNA, and **(F)** GC content.

For this study, the genome size of *A. pseudosieboldianum* was evaluated to be 558 Mb by *k*-mer distribution analysis (Supplementary Figure 1), yielding a total of 53.54 Gb bp raw data from the Illumina platform with a paired-end (PE) read length of 150 bp. The survey analysis showed that this genome had a high level of heterozygosity (1.10%) and repetition (76.84%) (Supplementary Table 1), indicating a complicated genome for *A. pseudosieboldianum*. We produced ∼30.37× coverage of approximately 20.97 Gb PacBio CCS long reads using single-molecule real-time (SMRT) sequencing technology on the PacBio Sequel II platforms and 100× coverage of approximately 68 Gb Hi-C paired-end reads (Supplementary Figure 2). After primary assembly, correction and polishing were conducted, the total length of the final assembly was 690.24 Mb with 287 contigs and a contig N50 length of 4.36 Mb, 44 scaffolds and a scaffold N50 length of 48.87 Mb, respectively. The number of contigs was lower than that in *A. yangbiense* and *A. truncatum*, indicating a high-quality *A. pseudosieboldianum* genome assembly. As a result, we obtained a high-quality assembly distributed across 13 chromosome-level pseudomolecules (Figure 2) and chromosomal lengths from 38.69 to 74.29 Mb representing 97.94% of the assembly. The size of the assembled genome was higher than the estimated genome size on the basis of survey analysis (∼558.49 Mb), which might be due to the high heterozygosity. Assessment of the integrity and quality of the assembled genome revealed that the total mapping rate was 99.43%. Then, Benchmarking Universal Single Copy Orthologs (BUSCO) results indicated that 1,588 complete conserved core plant genes (98.4% of the core genes) were found in the assembly genome (Supplementary Table 2), which was obviously higher than the values obtained in two recently released *A. yangbiense* and *A. truncatum* genomes sequenced by PacBio Sequel platforms. These results indicated that the genome assembly in the present study was accurate, and we obtained a chromosome-level reference genome of *A. pseudosieboldianum*.

### Repeat Sequences and Gene Annotation

In total, 530,503,700 bp of repetitive sequences were identified using homology-based and *de novo* predictions in the assembled genome of *A. pseudosieboldianum*, accounting for 76.84% of the genomic assembly (Supplementary Table 3). Among these repetitive sequences, transposable elements (TEs) were predominant (75.2%), whereas there were 48,300,502 bp of tandem repeat sequences, constituting 7% of the assembly (Supplementary Table 4). In addition, the majority of TEs were members of the long terminal repeat (LTR) retrotransposon family (397,284,964 bp, 57.55%) in the genome assembly, in which 130,179,606 (18.86%) bp of *Gypsy* subfamily accounted for the genome, followed by the *Copia* subfamily (154,672,142 bp, 22.41%) (Supplementary Table 5).

For protein-coding gene annotation of the *A. pseudosieboldianum* genome, three methods were used, including *ab initio* gene prediction, homology-based methods and RNA-Seq-aided strategies and finally the predictions were integrated. Approximately 29,679 protein-coding gene models were predicted and functionally annotated with an average gene length of 3,817.09 bp (Table 1 and Supplementary Table 6). The predicted gene number for *A. pseudosieboldianum* was slightly higher than that of *A. yangbiense* (28,320) and *A. truncatum* (28,438) (Table 1). The predicted gene contained an average coding sequence (CDS) length and average exon length of 1,179.31 and 283.76 bp, respectively (Supplementary Figure 3). In addition to protein-coding genes, we also identified 1,014 (0.011%) tRNAs, 1,515 (0.040%) rRNAs, 82 (0.002%) miRNAs and 297 (0.005%) snRNAs in the *A. pseudosieboldianum* genome (Supplementary Table 7). To investigate potential function, a sequence similarity search was performed with an *E*-value threshold of 1E-5. Among these identified protein-coding genes, 27,531 (92.76%) were annotated to at least one of the public databases, including the Integrated Resource of Protein Domains and Functional Sites (InterPro) database (22,819, 76.89%), Gene Ontology (GO) database (16,141, 54.39%), Kyoto Encyclopedia of Genes and Genomes (KEGG) database (27,376, 92.24%), Swiss-Prot protein database (20,440, 68.87%), Translated European Molecular Biology Laboratory (TrEMBL) database (27,413, 92.365), and NCBI non-redundant protein (NR) database (27,451, 92.49%) (Table 1, Supplementary Table 8 and Supplementary Figure 4). These results further supported the integrity of our genome.

**TABLE 1:** Summary of the genome assembly and annotation in *Acer* species.

Genomic features	*A. pseudosieboldianum*	*A. yangbiense*	*A. truncatum*
Raw bases of Sequel II (Gb)	20.97	64	76
Raw bases of Hic (Gb)	68	111.31	72.21
Genome size (Mb)	690.24	666	633.28
Number of scaffolds (bp)	44	280	34
Size of scaffolds (bp)	690,370,816	*	633,280,000
N50 of scaffolds (bp)	48,866,705	44,917,698	46,360,000
Number of contigs	287	562	1453
Size of contigs	690,249,316	665,887,899	628,840,000
N50 of contigs (bp)	5,760,274	5,479,097	773,170
Complete BUSCOs (%)	1,588 (98.4%)	1,375 (95.5%)	1,342 (93.2%)
GC content of the genome (%)	36.22%	35.96%	35.00%
Number of predicted protein-coding genes	29,679	28,320	28,438
Average gene length (bp)	3,817.09	3,880.55	3,457.58
Average CDS length (bp)	1,179.31	1,308.23	1,111.28
Average exon length (bp)	283.76	271.49	237.15
Number of tRNA	1,014	1,116	744
Number of rRNA	1,515	248	368
Number of miRNA	82	*	1,345
Number of snRNA	297	*	868
Repeat sequences (bp)	530,503,700 (76.84%)	452,810,000 (68.0%)	391,050,000 (61.75%)
Annotated to InterPro	22,819 (76.89%)	*	27,826 (97.84%)
Annotated to GO	16,141 (54.39%)	21,833 (77.10%)	25,432 (89.43%)
Annotated to KEGG	27,376 (92.24%)	9,666 (34.10%)	21,276 (74.81%)
Annotated to Swiss-Prot	20,440 (68.87%)	16,596 (58.6%)	21,289 (74.86%)
Annotated to NR	27,451 (92.49%)	26,235 (92.6%)	27,202 (95.65%)

*Indicates data were not shown in the original articles.

### Evolution of the *A. pseudosieboldianum* Gene Family

**FIGURE 3:**
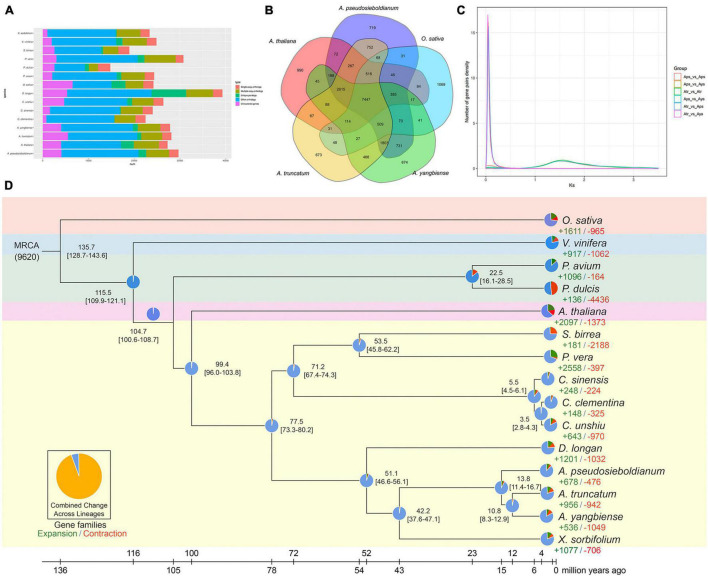
Gene family, phylogenetic analysis, and 4dTV and *K*_s_ distribution of *A. pseudosieboldianum* and *Acer* species. **(A)** Number of genes in various plant species, showing a high gene number for *A. pseudosieboldianum* compared with other species. **(B)** Venn diagram of the gene family between *A. pseudosieboldianum*, *A. yangbiense*, *A. truncatum*, *A. thaliana*, *Populus trichocarpa*, and *O. sativa*. **(C)** Distribution of *K*_s_. **(D)** Species tree on the basis of 347 single-copy orthologs from 16 plant species.

To understand the evolution of the *A. pseudosieboldianum* genome, we performed a comparative genomic analysis among different species. To identify the gene families, the analysis of sequence similarity-based clustering was performed. The analysis of orthologous and paralogous genes among 15 species revealed that 2,087 single-copy orthologs and 1,932 unique paralogous genes were specific to *A. pseudosieboldianum* (Figure 3A and Supplementary Table 9). In total, 29,679 genes of *A. pseudosieboldianum* were clustered into 16,666 gene families, of which 7,395 were shared with the other five species, and 772 gene families appeared to be unique to *A. pseudosieboldianum* (Figures 3A,B and Supplementary Table 10). The number of unique gene families in *A. pseudosieboldianum* was similar to that of the other two *Acer* species, indicating a close evolutionary relationship between them. Furthermore, for three *Acer* species, 12,374 gene families were common, while the unique gene families were 868, 777, and 819 in *A. pseudosieboldianum*, *A. yangbiense*, and *A. truncatum*, respectively (Supplementary Figure 5). The phylogenetic tree was constructed using 560 strictly single-copy gene sets (ortholog families) from 15 plant species (see Section “Materials and Methods”) with fully sequenced genomes, including 10 sapindales species, two rosales species, two other plants and *O. sativa* as a outgroup. Phylogenomic analysis revealed that *A. pseudosieboldianum* was most related to the ancestor of *A. yangbiense* and *A. truncatum*, with an estimated divergence time of ∼13.8 (11.4–16.7) million years ago (Mya) (Figure 3D). Three *Acer* species and *X. sorbifolium* diverged from their common ancestor approximately 42.2 (37.6–47.1) Mya, while the split of *P. vera* and *S. birrea* was estimated to have occurred 53.5 (45.8–62.2) Mya. In addition, the divergence of Sapindales species with *A. thaliana* was estimated to have occurred at 99.4 (96.0–103.8) Mya.

The significant expansion or contraction of each gene family across species was evaluated, and the results showed that out of 16,229 identified gene families in the *A. pseudosieboldianum* genome, 678 had significantly expanded (*p* < 0.05), and 476 had contracted (*p* < 0.05). GO enrichment analysis suggested that *A. pseudosieboldianum* expanded gene families revealed a significant enrichment in transferase activity, single-organism metabolic process, tetrapyrrole binding, heme binding, potassium ion transmembrane transport, among others (Supplementary Table 11). KEGG pathway analysis of these expanded gene families showed that they were mainly enriched in the biosynthesis of siderophore group non-ribosomal peptides, sesquiterpenoid and triterpenoid biosynthesis, alpha-linolenic acid metabolism and linoleic acid metabolism (Supplementary Table 12).

### Analyses of Genome Synteny and Whole-Genome Duplication

**FIGURE 4:**
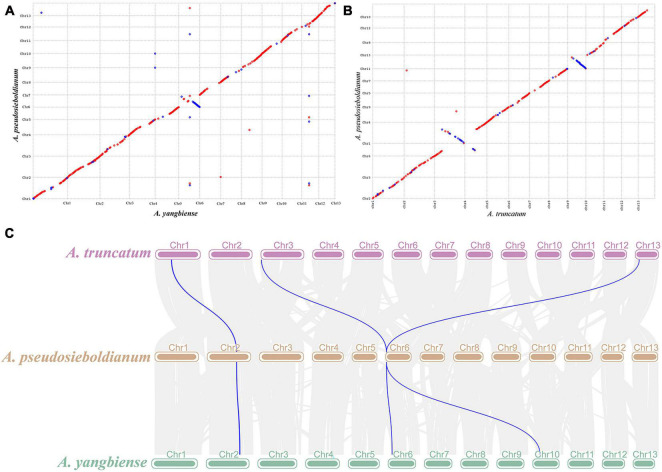
Synteny analysis of *A. pseudosieboldianum*, *A. yangbiense* and *A. truncatum*. **(A)** Dot plots of syntenic blocks between *A. pseudosieboldianum* and *A. yangbiense*. **(B)** Dot plots of syntenic blocks between *A. pseudosieboldianum* and *A. truncatum*. The red line represents sequence forward matching, and the blue line represents reverse complementary matching. **(C)** Blocks with syntenic genes among *A. pseudosieboldianum*, *A. yangbiense*, and *A. truncatum*. The blue line shows an example of two syntenic blocks.

To further identify genome synteny, the synteny blocks within the *A. pseudosieboldianum* genomes and related *Acer* species were examined. The collinearity analysis found that a total of 33,631 and 33,791 collinear gene pairs from 579 and 508 collinear blocks were detected between *A. pseudosieboldianum* and *A. yangbiense* or *A. truncatum*, respectively, which suggested a high degree of conserved gene order in *A. pseudosieboldianum* and two *Acer* species (Figures 4A,B). In particular, the syntenic blocks identified in *A. pseudosieboldianum* and *A. yangbiense* were higher than those between *A. pseudosieboldianum* and *A. truncatum*, indicating a similar evolutionary history between them (Figure 4C). For example, one gene (*rna-Apse002T0174700.1*) on chr2 in *A. pseudosieboldianum* was found to share origins with chr1 in *A. truncatum* and chr2 in *A. yangbiense*, while two genes (*rna-Apse006T0011300.1* and *rna-Apse006T0012000.1*) located on Chr6 in *A. pseudosieboldianum* shared origins with Chr3 and Chr13 in *A. truncatum* and Chr6 and Chr10 in *A. yangbiense*. These results indicated a high ancestral collinearity between these *Acer* species.

Whole-genome duplication is a crucial factor during species evolution, and it can improve species adaptability and resistance. The distributions of synonymous substitutions per synonymous site (*K*_s_) of collinear gene pairs were further used to confirm the WGD events. The *K*_s_ distribution revealed that a significant main peak was identified at approximately around 1.50 units (Figure 3C), which is consistent with the results of *A. truncatum* shown in Figure 3A (Ma et al., 2020). This indicates that an ancient duplication occurred in *A. pseudosieboldianum*, similar to most plant species, which means γ event shared by all core eudicots (Jiao et al., 2011; Mcgrath et al., 2014). And no smaller peaks were found which indicate no recent WGDs, similar to *A. yangbiense* (Yang et al., 2019) and *A. truncatum* (Ma et al., 2020).

### Transcription and Metabolism for Leaf Pigmentation Regulation in *A. pseudosieboldianum*

**FIGURE 5:**
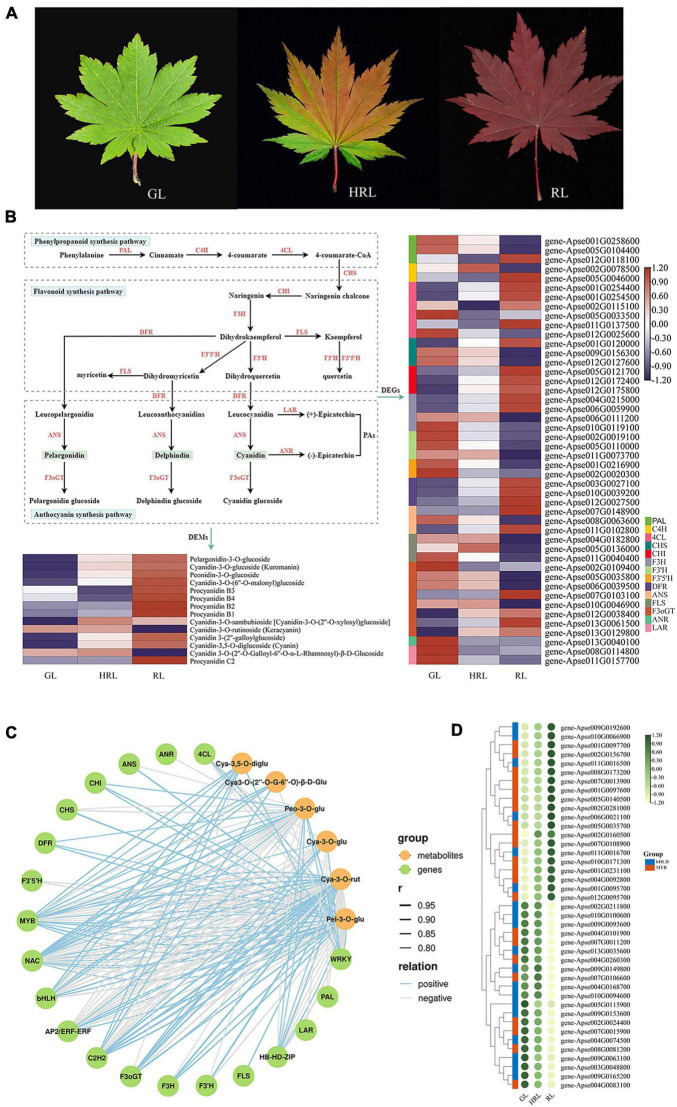
Comparative transcriptomic analysis of genes involved in anthocyanin biosynthesis during leaf color change. **(A)** Photographs of different leaf colors of *A. pseudosieboldianum*. GL, green leaf; HRL, half-red leaf; RL, red leaf. **(B)** The biosynthesis pathways of flavonoids, anthocyanin and flavonoid and the expression analysis of differentially expressed genes and differentially expressed metabolites. **(C)** Correlation network of metabolite-related genes involved in anthocyanin biosynthetic pathways. *r* represents the Pearson correlation coefficient; relation represents the correlation, including positive (*r* > 0.8) and negative correlations (*r* < –0.8). **(D)** Heatmap showing the differential expression of MYB and bHLH transcription factors according to the transcriptome data from leaves of different colors.

*Acer pseudosieboldianum* has been confirmed to possess relatively higher ornamental value and stress tolerance than many other *Acer* species. To better understand how *A. pseudosieboldianum* displays extremely red leaf color in autumn at the molecular level, an experiment with three different developmental stages of leaf, green leaf (GL), half-red leaf (HRL), and red leaf (RL), was conducted combining transcriptomics and metabolomics technologies (Figure 5A). A total of 5,634 DEGs were identified in the GL vs. RL comparison, consisting of 2,684 upregulated and 2,950 downregulated genes (Supplementary Table 13), and 113 (58 upregulated and 55 downregulated) differentially expressed metabolites (DEMs) were also simultaneously found based on the variable importance in projection (VIP) >1 and fold change >2 or <0.5 (Supplementary Table 14). The main enriched GO terms were secondary metabolic process (GO:0019748) and tetrapyrrole binding (GO:0046906) (Supplementary Figure 6), and the top enriched KEGG pathways were metabolic pathways and biosynthesis of secondary metabolites (Supplementary Figure 7). In the comparison of GL vs. HRL, a total of 1,430 DEGs (487 upregulated and 943 downregulated) and 47 DEMs (27 upregulated and 20 downregulated) were identified. Of the GO enrichment terms, the highest ranking was cell wall organization (GO:0071555) and plasma membrane part (GO:0044459), and these DEGs were also enriched with KEGG pathways (Supplementary Figures 8, 9). A comparison between HRL vs. RL revealed 3,927 DEGs, with 1,823 upregulated and 2,104 downregulated genes, and 94 DEMs, including 36 upregulated and 58 downregulated metabolites (Supplementary Tables 13, 14). Simultaneously, 527 genes were identified as common DEGs in the three comparisons (Supplementary Figure 10).

We investigated the key structural genes involved in phenylpropane, flavonoids and anthocyanins during leaf color change in *A. pseudosieboldianum*. The results revealed that 46 DEGs were known to be related to anthocyanin biosynthesis pathways, among which the majority of genes encoding C4H, 4CL, CHI, F3H, DFR, and F3oGT enzymes were upregulated in GL vs. RL comparison (Figure 5B and Supplementary Table 15). Furthermore, all genes encoding CHI and F3H exhibited high expression in RL compared with GL and HRL. In addition to *ANR*, the other genes were demonstrated to be a multigene family. In addition to some structural genes, the biosynthesis of anthocyanin during leaf color change was influenced by some regulators. In total, in the three group comparisons (GL vs. HRL, GL vs. RL, and HRL vs. RL), we identified 800 transcription factors contributed by DEGs, among which the top seven TFs were NAC (71), bHLH (62), MYB (62), C2H2 (51), AP2/ERF-ERF (42), WRKY (37), and HB-HD-ZIP (30), revealing that they might be related to anthocyanin accumulation with leaf color change (Supplementary Table 16).

From the principal component analysis shown in Supplementary Figure 11, the obvious distinction of metabolites was found from different sample groups, and these metabolites can be used for next step of metabolomics analysis. Accordingly, we identified 333 secondary metabolite compounds that could be grouped into 7 classes: phenolic acids (150), flavonoids (119), tannins (37), lignans and coumarins (15), terpenoids (6), alkaloids (3), and other substances (3), among which 14 DEMs belonging to anthocyanin were detected and showed significantly higher expression in RL than in GL and HRL (Supplementary Table 17). Interestingly, the anthocyanin identified in this study mainly consists of pelargonidin, cyanidin, peonidin and procyanidin, and the content of pelargonidin always maintained a relatively high expression level in red leaves (Figure 5B), indicating that pelargonidin may play a key role in leaf color formation in *A. pseudosieboldianum*. The accumulation pattern of anthocyanin agreed with the expression of genes encoding key enzymes in the anthocyanin pathway.

Using liquid chromatography mass spectrometry (LC-MS), we observed that the identified DAMs were significantly enriched in the phenylpropanoid, flavonoid, and anthocyanin synthesis pathways, which is similar to the results obtained from transcriptome data sets, indicating that the accumulation pattern of metabolites was regulated by differential gene expression. To elucidate the correlation between the transcriptome and metabolome during leaf color change, we constructed a gene-to-metabolite network with a correlation coefficient of *r* > 0.8 or <−0.8, and the correlation network was further visualized using OmicStudio tools^7^. The results of the correlation between the metabolic and transcriptomic analysis showed that a total of 6,534 DEGs were significantly correlated with 146 DAMs, accounting for 43.84% of the identified metabolites. In particular, we observed that the identified structural genes and key transcription factors were significantly correlated with six anthocyanins, including pelargonidin-3-*O*-glucoside, cyanidin-3-*O*-rutinoside (Keracyanin), cyanidin-3-*O*-glucoside (Kuromanin), cyanidin 3-*O*-(2′′-*O*-Galloyl-6′′-*O*-α-L-rhamnosyl)-β-D-Glucoside, cyanidin-3,5-*O*-diglucoside and peonidin-3-*O*-glucoside (Figure 5C and Supplementary Tables 18, 19). Some crucial enzyme-encoding DEGs involved in anthocyanin biosynthesis, such as *CHI* (EC:5.5.1.6), *DFR* (EC:1.1.1.219), and *F3oGT* (EC:1.1.1.219), were positively related to the accumulation of six anthocyanins. Additionally, in this network, metabolites of cyanidin-3-*O*-rutinoside and pelargonidin-3-*O*-glucoside were strongly correlated with the expression of transcription factors, especially MYB and bHLH TFs, indicating that these two anthocyanins play a significant role in leaf color change in *A. pseudosieboldianum*. To gain further insight into the expression patterns and potential function of the R2R3-MYB and bHLH TFs, the expression patterns of these two TFs were investigated (Figure 5D and Supplementary Table 19). In the heatmap, numerous members of R2R3-MYB TFs were highly expressed at the red-leaf (RL) stage, showing a significant positive regulatory function. In contrast, the majority of bHLHs had much lower expression in RL, indicating a negative regulatory function. These results revealed that anthocyanin biosynthesis genes and regulators might be involved in leaf color change in *A. pseudosieboldianum*.

### Teosinte Branched1, Cycloidea, and Proliferating Cell Factors Family Genes

**FIGURE 6:**
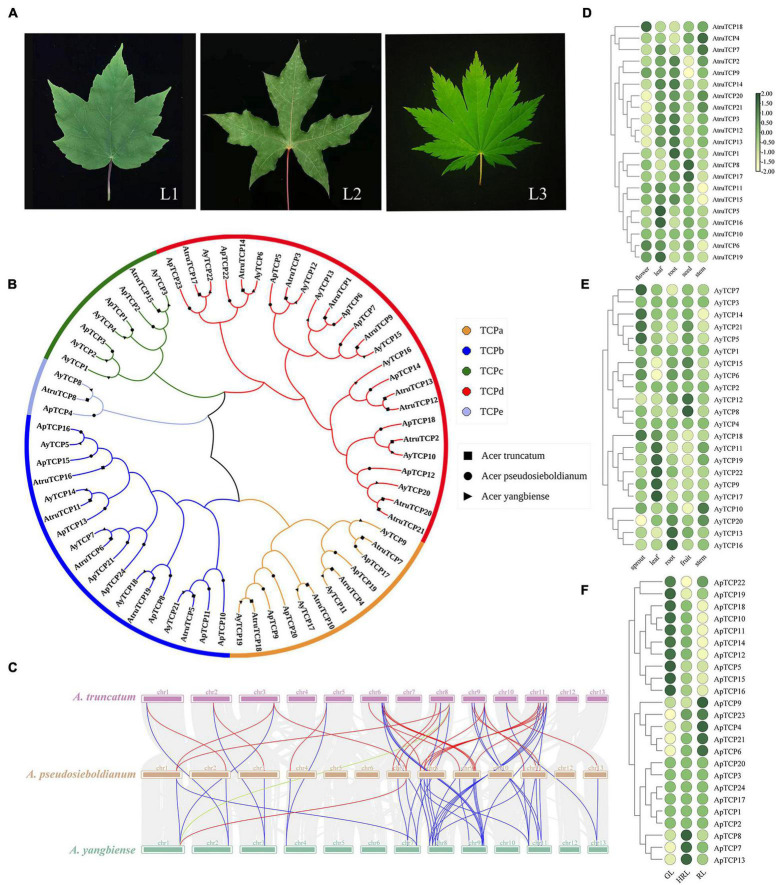
Teosinte branched1, cycloidea, and proliferating cell factors (TCP) gene family in *A. pseudosieboldianum*, *A. yangbiense*, and *A. truncatum*. and expression analysis of TCP genes in different tissues. **(A)** Photographs of leaf shape in *A. yangbiense*, *A. truncatum*, and *A. pseudosieboldianum*. L1 represents the mature leaf in *A. yangbiense*; L2 represents the mature leaf in *A. truncatum*; L3 represents the mature leaf in *A. pseudosieboldianum*. **(B)** Phylogenetic tree of TCP genes from *A. pseudosieboldianum* (24 genes), *A. yangbiense* (22 genes), and *A. truncatum* (21 genes). **(C)** Synteny analysis of TCP genes in *A. pseudosieboldianum*, *A. yangbiense*, and *A. truncatum*. Gray lines in the background indicate collinear blocks between *A. pseudosieboldianum*, *A. yangbiense*, and *A. truncatum*, whereas red lines highlight syntenic *ApTCP* gene pairs, blue lines highlight syntenic *AtruTCP* gene pairs, and green lines highlight syntenic *AyTCP* gene pairs. Expression analysis of TCP genes in different tissues of *A. truncatum*. **(D)** Heatmap showing the expression level of TCP family in different tissues in *A. truncatum*. **(E)** Heatmap showing the expression level of TCP family in different tissues in *A. yangbiense*. **(F)** Heatmap showing the expression level of TCP family in different tissues in *A. pseudosieboldianum.*

In addition to varied leaf colors, *Acer* species also have a variety of leaf shapes, ranging from non-split to split leaves and single to compound leaves (Figure 6A). To examine the molecular mechanism involved in leaf shape variations, we first performed a genome-wide search of putative TCP genes in both *A. yangbiense*, *A. truncatum* and the assembled genome of *A. pseudosieboldianum*. A total of 67 TCP genes were identified, 24 from *A. pseudosieboldianum*, 22 from *A. yangbiense* and 21 from *A. truncatum*, which is comparable to the number in *A. thaliana* TCP (24) families, showing relatively high conservation in dicotyledonous plants (Supplementary Table 20). To further elucidate the classification relationship of TCP proteins in these three *Acer* species, a neighbor-joining phylogenetic tree was constructed based on multiple alignment of the TCP protein sequences. The 67 TCP proteins of all three *Acer* species were further divided into five groups: TCPa (12 genes), TCPb (18 genes), TCPc (3 genes), TCPd (8 genes), and TCPe (26 genes) (Figure 6B). According to the phylogenetic tree, the majority of the TCPs were classified into the same clade with one member for each *Acer* species, confirming the reliability of our phylogenetic tree. However, some of the TCPs were specific, such as *AyTCP16*, *ApTCP12*, *ApTCP10*, *ApTCP24*, and *AtruTCP16*, indicating that these TCPs might be related to the variation in leaf morphology for each *Acer* species. Furthermore, to understand the evolutionary mechanisms of the TCP genes, a syntenic analysis of the TCP genes in three *Acer* species (*A. yangbiense*, *A. truncatum*, and *A. pseudosieboldianum*) was performed. The syntenic analysis indicated that all TCP gene pairs we identified were mainly distributed on chromosomes 1, 9, and 9 in *A. yangbiense*, *A. truncatum*, and *A. pseudosieboldianum*, respectively (Figure 6C). In particular, *A. pseudosieboldianum* and *A. truncatum* displayed the highest homology, which was consistent with the results of genome syntenic analysis. To understand the function of TCPs in organ development, we analyzed the tissue-specific expression profiles of TCP genes in three *Acer* species based on previous RNA-seq data. For *A. truncatum*, the expression levels of some *AtruTCP* genes were differentially varied in the five organs, including the flower, leaf, root, seed, and stem, revealing an obvious functional divergence of TCP genes during *A. truncatum* development (Figure 6D and Supplementary Table 21). Particularly, for *A. yangbiense*, the TCPa subfamily, including *AyTCP9*, *AyTCP11*, *AyTCP17*, and *AyTCP19*, maintained significantly high expression levels in leaves and relatively low levels in other tissues, indicating that they might play a key role in leaf development (Figure 6E and Supplementary Table 22). Furthermore, in *A. pseudosieboldianum*, *ApTCP4*, *ApTCP6*, *ApTCP9*, *ApTCP21*, and *ApTCP23* were exclusively and highly expressed in mature leaves (RL) and presented low expression levels in early young leaves (GL), implying their specific roles in leaf development during the whole life span of leaves in *A. pseudosieboldianum* (Figure 6F and Supplementary Table 23).

## Discussion

*Acer pseudosieboldianum* is an evolutionarily and ecologically important ornamental wood species which is widely used as a prospective colored-leaf plant species for commercial purposes. Here, we obtained the chromosome-scale genome assembly of *A. pseudosieboldianum* (∼690 Mb) based on PacBio sequel II sequencing data with long HiFi reads, which was the highest quality compared with the recently reported *Acer* species and the family Aceraceae genome, with a contig N50 size of 5.7 Mb and a genome completeness of 98.6% in terms of the BUSCO assessment, indicating a high genome integrity. The availability of the *A. pseudosieboldianum* genome sequence is also of great importance because it is an endemic species in northeast China, and occupying a unique evolutionary position among *Acer* species. Therefore, the high-quality chromosome-level assembly obtained herein provides useful genetic information for accelerating the progress of marker-assisted breeding, functional gene discovery and comparative genomics across the Aceraceae family.

### Genome Quality of *A. pseudosieboldianum*

Previous studies have revealed that the proportion of heterozygous and repetitive sequences was the key challenge for genome assembly and subsequent analyses (Chen et al., 2021). In our study, the estimated heterozygosity in *A. pseudosieboldianum* (1.1%) was relatively high compared with *A. yangbiense* (0.19%) (Yang et al., 2019) but slightly lower than that of *A. truncatum* (1.12%) (Ma et al., 2020). Furthermore, the proportion of repeat sequences of *A. pseudosieboldianum* (76.84%) was significantly higher than that of *A. yangbiense* (68.75%) (Yang et al., 2019) and *A. truncatum* (61.75%) (Ma et al., 2020), indicating a complex genome of *A. pseudosieboldianum*. We inferred that this difference might be related to the biological characteristics of heterodichogamy (such as protandry, protogyny, and duo-dichogamy) and the wide wind pollination in *Acer* species. In particular, in previous investigations, we found both bisexual and unisexual flowers during the occurrence and development of flower organs in *A. pseudosieboldianum*, further supporting the above results. To overcome the challenge of assembling the complex *A. pseudosieboldianum* genome, assembly strategies using high-precision long-read (HiFi) and Hi-C sequence data were employed to obtain the final chromosome-level reference genome (∼ 690 Mb). The contig N50 size (5.7 Mb) and genome completeness (98.6%) based on BUSCO results for *A. pseudosieboldianum* was significantly higher than those of a recent assembly of *A. yangbiense* (Yang et al., 2019) (contig N50 of 5.4 Mb and genome completeness of 95.5%), *A. truncatum* (Ma et al., 2020) (contig N50 of 0.7 Mb and genome completeness of 93.2%), *A. catalpifolium* (contig N50 of 0.7 Mb, genome completeness of 93.3%) (Yu et al., 2021), *Acer negundo* (contig N50 of 0.7 Mb, genome completeness of 97.7%) and *Acer saccharum* (contig N50 of 0.95 Mb, genome completeness of 97.4%) (McEvoy et al., 2021). These results further revealed that our assembled genome represented the highest-level reference genome at present for Aceraceae family species.

### Ancient Origins of *A. pseudosieboldianum*

High-quality genomic sequences are essential for studying the gene function, evolutionary history, and genetic map construction of a species (Chaw et al., 2019). Species in Aceraceae family are critically important maple trees and consist of the *Acer* and *Dipteronia* genera with more than 200 species, and the majority of species in this family belong to the genus *Acer* (∼198 species) (Xu, 1998; Ma et al., 2020). A recently assembled genome of the Aceraceae family contains only *A. yangbiense* (Yang et al., 2019) and *A. truncatum* (Ma et al., 2020), and the evolutionary history of the Aceraceae family is still unclear because of the lack of available genome information. The taxonomic status of Aceraceae species is still controversial. In particular, many taxonomists believe that the present Aceraceae family should be divided into Sapindaceae in terms of the angiosperm phylogeny group (APG) system, and Aceraceae and Sapindaceae are largely considered paraphyletic groups (Harrington et al., 2005). However, a previous study has suggested listing Aceraceae as a separate family that is itself monophyletic (Wu et al., 2008). In our study, 347 single-copy orthologous gene families obtained from gene family cluster analysis of 16 species were employed to construct the phylogenetic tree. Our results showed that a total of 10 candidate species from Sapindales were clearly clustered into the same evolutionary branch at the genome level, with similar evolutionary characteristics. Particularly, *A. pseudosieboldianum* showed the closest relationship to the ancestor of *A. yangbiense* and *A. truncatum*, revealing their closer relationship. In addition, the genera *Xanthoceras* and *Acer* were the most related, which is consistent with published research (Gadek et al., 1996; Wu et al., 2008). These results unambiguously demonstrate that the Aceraceae family should be incorporated into the Sapindaceae according to genome evolutionary analysis, which is in agreement with the APG taxonomy system.

### The Genes Related to Leaf Color Change

As a representative of the *Acer* species, *A. pseudosieboldianum* possesses bright red leaf colors during late autumn and has the potential to become an ideal colored-leaf tree for ornamental and horticultural usages. Elucidation of the coordination mechanism of gene metabolites involved in leaf color formation would contribute to the identification of leaf color-related genes in *A. pseudosieboldianum*. Anthocyanins, the key metabolites of leaf/fruit color formation in plants, have antioxidant, antiaging and anti-inflammatory functions, and their biosynthesis pathways have been widely investigated in many plants (Zhang et al., 2017; Zhou et al., 2019; Li et al., 2021b). Previous studies have demonstrated that the formation of leaf color in plants is mainly regulated by anthocyanins, which could influence pigmentation and relative gene expression (Jia et al., 2020; Qiu et al., 2020). It has been shown that six anthocyanidins, pelargonidin, malvidin, peonidin, cyanidin, delphinidin, and petunidin, are common in leaf/fruit color changes (Sulc et al., 2017; Li et al., 2021c). In our study, 14 differential anthocyanins were identified in the leaves of *A. pseudosieboldianum* and mainly contained three categories of pelargonidin, cyanidin and peonidin. Additionally, almost all compounds showed significantly high expression in red leaves (RL), especially pelargonidin, and thus, they may be sources of red leaf formation. Similar results have also been confirmed in previous investigations (Li et al., 2021a; Zhang et al., 2021). Furthermore, some key structural genes involved in anthocyanin biosynthesis were also found based on RNA-seq profiling, and the majority of ANS and DFR were significantly upregulated, indicating a greater effect on anthocyanin accumulation. Anthocyanin biosynthesis was also regulated by transcription factors such as MYB, bHLH, WD40 and WRKY, of which the MBW complex is considered a key regulator. In the present study, the top seven transcription factors, NAC, MYB, C2H2, AP2/ERF, bHLH, WRKY, and HB-HD-ZIP, showed significantly different expression levels between green and red leaves. We speculated that they might be candidate regulators of anthocyanin accumulation in leaf color changes in *A. pseudosieboldianum*. We also constructed a gene-metabolite correlation network, and the results showed that the metabolites cyanidin-3-*O*-rutinoside and pelargonidin-3-*O*-glucoside were strongly correlated with structural genes and several transcription factors, including MYB, C2H2, bHLH, and NAC, in anthocyanin biosynthesis pathways. These results further verified the roles of key genes and metabolites involved in anthocyanin accumulation during leaf color changes. In addition, several members of R2R3-MYB TFs were highly expressed at the red-leaf (RL) stage, while the majority of bHLHs had much lower expression in RL. This indicates that R2R3-MYB has a significant positive regulatory function, whereas bHLHs have a negative regulatory function. It has been observed that R2R3-MYB plays a central role in the regulation of anthocyanin biosynthesis, while bHLHs mainly plays a negative regulatory role in anthocyanin biosynthesis (Pu et al., 2021).

### The Teosinte Branched1, Cycloidea, and Proliferating Cell Factors Related to Leaf Shape Change

Leaf shape is a complex trait controlled by multiple genes and is of great significance for plant photosynthetic energy storage and resistance to stress (Testone et al., 2019; He P. et al., 2020). Teosinte branched1, cycloidea, and proliferating cell factors (TCPs), belonging to the bHLH transcription factor family, are widely involved in leaf morphology regulation (Aggarwal et al., 2010; Zhao M. L. et al., 2020; Lan et al., 2021). Previous studies have confirmed that TCP transcription factors widely participate in the regulation of flower shape, leaf shape, and stem and root development and have been identified in many plant species (Wang et al., 2019; He J. et al., 2020; Wen et al., 2020). Leaf shape in the Aceraceae family differs among different species, showing different variations, such as trifoliate, pinnate and lobed leaves. However, to date, few studies have focused on variations of leaf shape, and the identification of TCP gene families is still unclear for *Acer* species. Herein, using whole-genome sequence data, we comprehensively conducted a genome-wide identification of TCP genes in *A. pseudosieboldianum*, *A. yangbiense*, and *A. truncatum* to understand the regulation of leaf shape development. As indicated by gene family analysis, 24, 22, and 21 TCPs were identified in *A. pseudosieboldianum*, *A. yangbiense*, and *A. truncatum*, respectively, revealing obvious differences that may be due to gene loss or duplication events during the evolution of these three *Acer* species. Previous studies indicated that the *AtTCP24* (*AT1G30210.1*) gene regulated the leaf development, leaf morphogenesis and cell differentiation of *A. thaliana* (Efroni et al., 2008; Koyama et al., 2010; Wang et al., 2015). In present study, *AyTCP9*, *AyTCP11*, *AyTCP17*, and *AyTCP19* were significantly expressed in leaves compared with other tissues of *A. yangbiense*, and these four genes showed the highest degree of orthology with *AT1G30210.1* (Supplementary Table 20), suggesting that they might be involved in leaf shape developmental regulation and thus could be used as key candidate genes to increase the leaf differentiation in *A. yangbiense*. Furthermore, five key genes, *ApTCP4*, *ApTCP6*, *ApTCP9*, *ApTCP21*, and *ApTCP23*, displayed high expression levels in mature leaves of *A. pseudosieboldianum*. The protein sequences of *ApTCP9* and *ApTCP21* were highly similar with *AT1G30210.1* (Supplementary Table 20). Thus, it is speculated that *ApTCP9* and *ApTCP21* may specifically regulate the leaf development and morphogenesis in *A. pseudosieboldianum*. Taken together, these results suggest that these genes may have a potential regulatory effect on leaf development.

## Data Availability Statement

The assembled *A. pseudosieboldianum* genome has been deposited in the Genome Warehouse in National Genomics Data Center (NGDC) (https://ngdc.cncb.ac.cn/) under accession number PRJCA006356. Third-generation transcriptomic data have been deposited in NGDC under accession number PRJCA006369. Transcriptomic data have been deposited in the SRA database of NCBI under accession number PRJNA736515.

## Author Contributions

XL, KC, XP, and ZH was a major contributor in writing the manuscript. SZ, AS, YX, CZ, RH, and RG contributed to plant sample collection, DNA/RNA preparation, library construction, and sequencing. XL and KC worked on genome assembly and annotation. MT and RS conducted transcriptome analysis and identified functional genes involved in leaf color formation. XL and KC analyzed the gene family and constructed the evolutionary tree. XZ conceived of the study, participated in its design and data interpretation, and revised the manuscript critically. All authors contributed to the article and approved the submitted version.

## Conflict of Interest

The authors declare that the research was conducted in the absence of any commercial or financial relationships that could be construed as a potential conflict of interest.

## Publisher’s Note

All claims expressed in this article are solely those of the authors and do not necessarily represent those of their affiliated organizations, or those of the publisher, the editors and the reviewers. Any product that may be evaluated in this article, or claim that may be made by its manufacturer, is not guaranteed or endorsed by the publisher.

## Footnotes

1
https://pacbio.secure.force.com/SamplePrep

2
http://www.timetree.org/

3
https://github.com/StoreyLab/qvalue

4
https://github.com/tanghaibao/mcscan

5
https://www.ncbi.nlm.nih.gov/

6
https://www.arabidopsis.org/

7
https://www.omicstudio.cn/tool
