# Supplementary material for: Chromosome-Level Genome Assembly for Acer pseudosieboldianum and Highlights to Mechanisms for Leaf Color and Shape Change
Source: Front Plant Sci. 2022 Mar 3;13:850054. doi: 10.3389/fpls.2022.850054 (PMC8927880; doi:10.3389/fpls.2022.850054)
Supplement: Supplementary file 1 [file Data_Sheet_1.docx]

**Chromosome-level Genome Assembly of *Acer pseudosieboldianum* Highlights the Mechanisms for Leaf Color and Shape Change**

**Supplementary Figures**


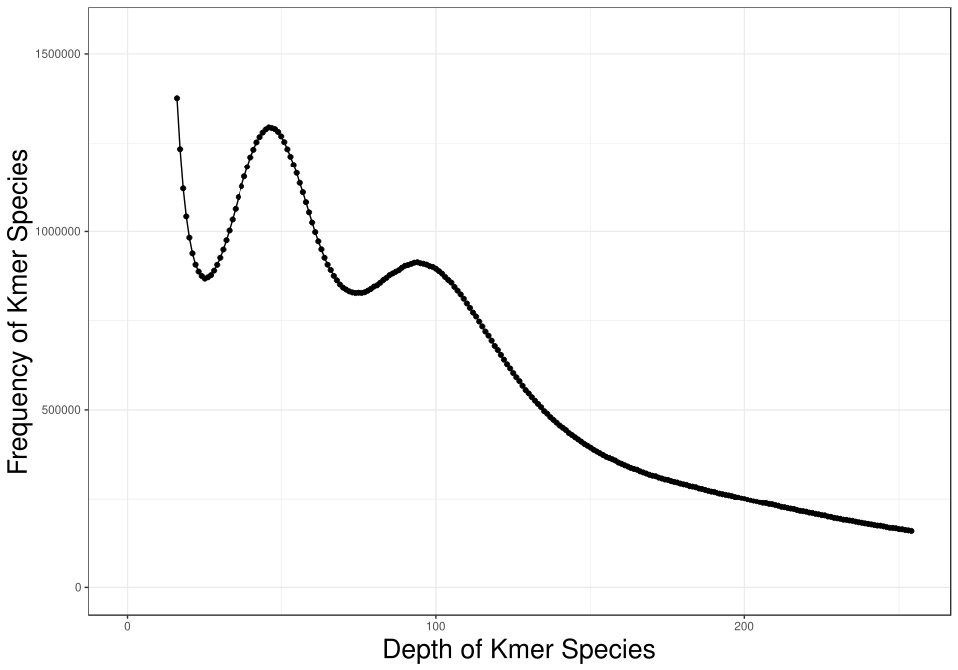


**Figure S1.** 15-mer analysis to estimate the [*A. pseudosieboldianum*](http://db.kib.ac.cn/CNFlora/SearchResult.aspx?CPNI=CPNI-111-20623) genome size. The x-axis and y-axis indicate the 15-mer number and frequency of kmer, respectively.


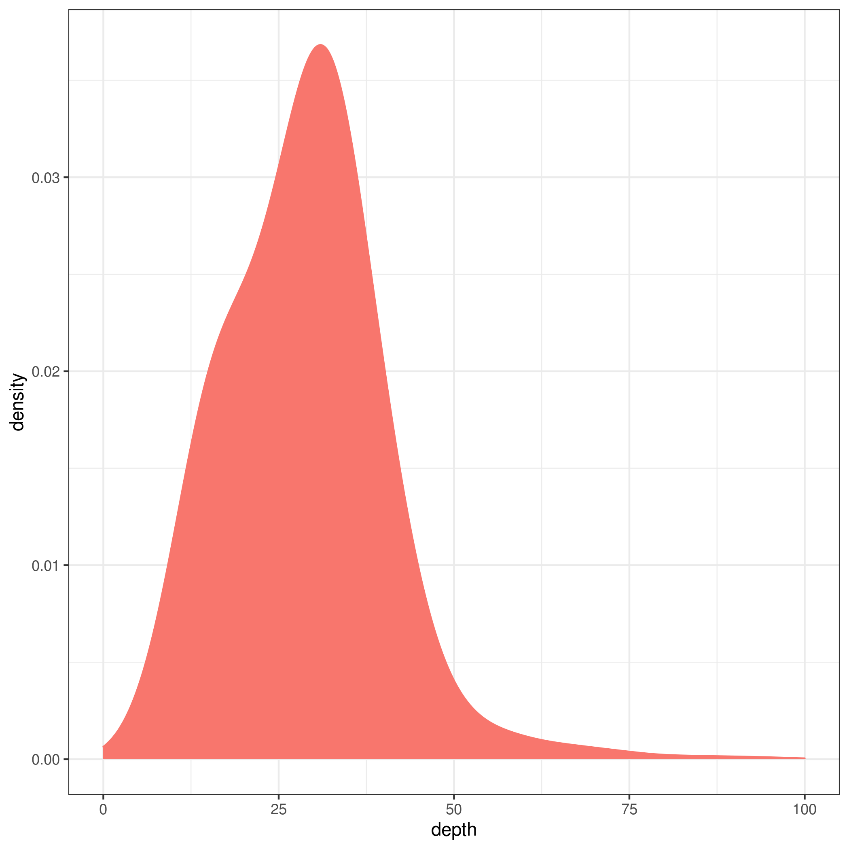


**Figure S2.** Sequencing depth distribution of the assembled *A. pseudosieboldianum* genome.


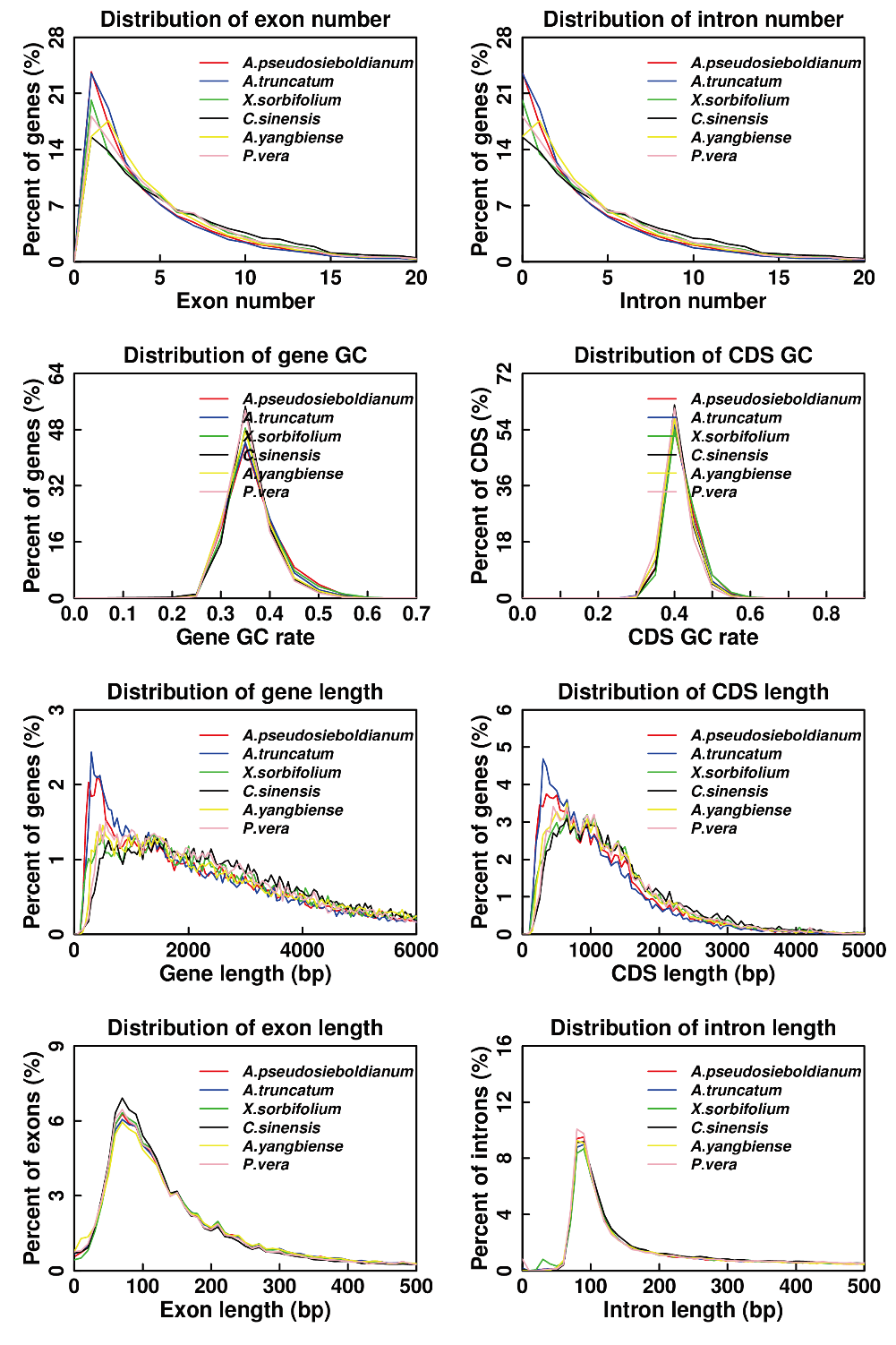


**Figure S3.** Cross-species comparisons of exon number, intron number, gene GC, CDS GC, gene length, CDS length, exon length and intron length distribution in six species.


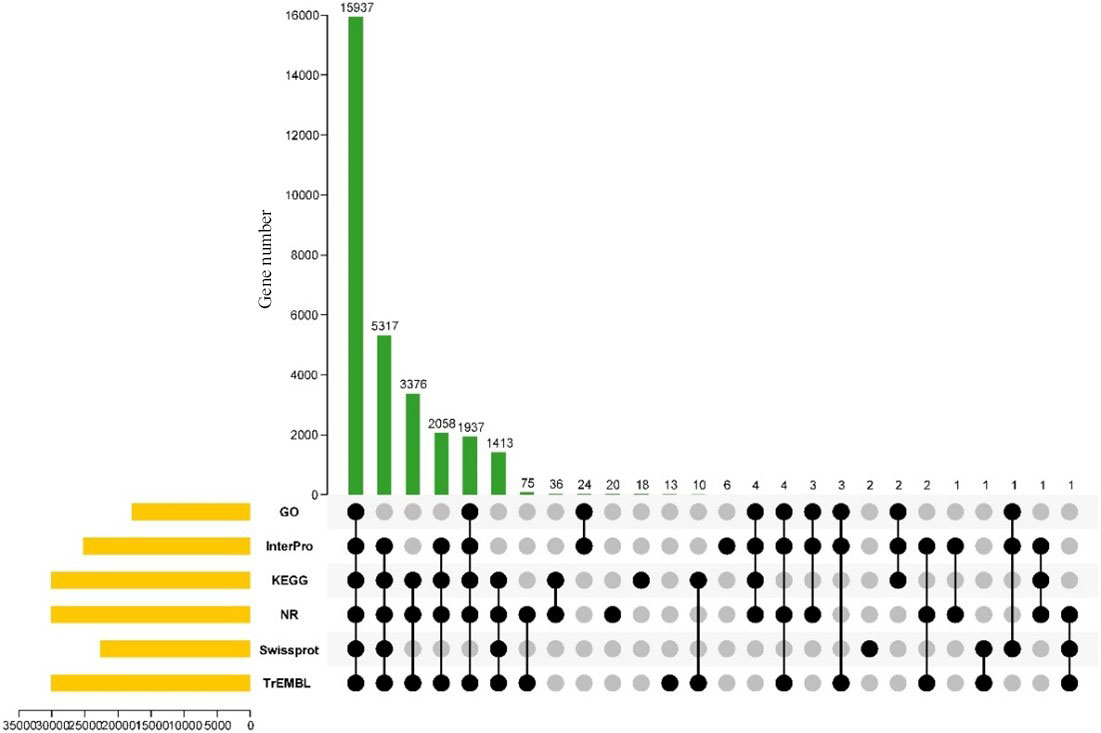


**Figure S4.** Upset plot of genes annotated in GO, InterPro, KEGG, NR, Swissprot and TrEMBL database in *A. pseudosieboldianum.*


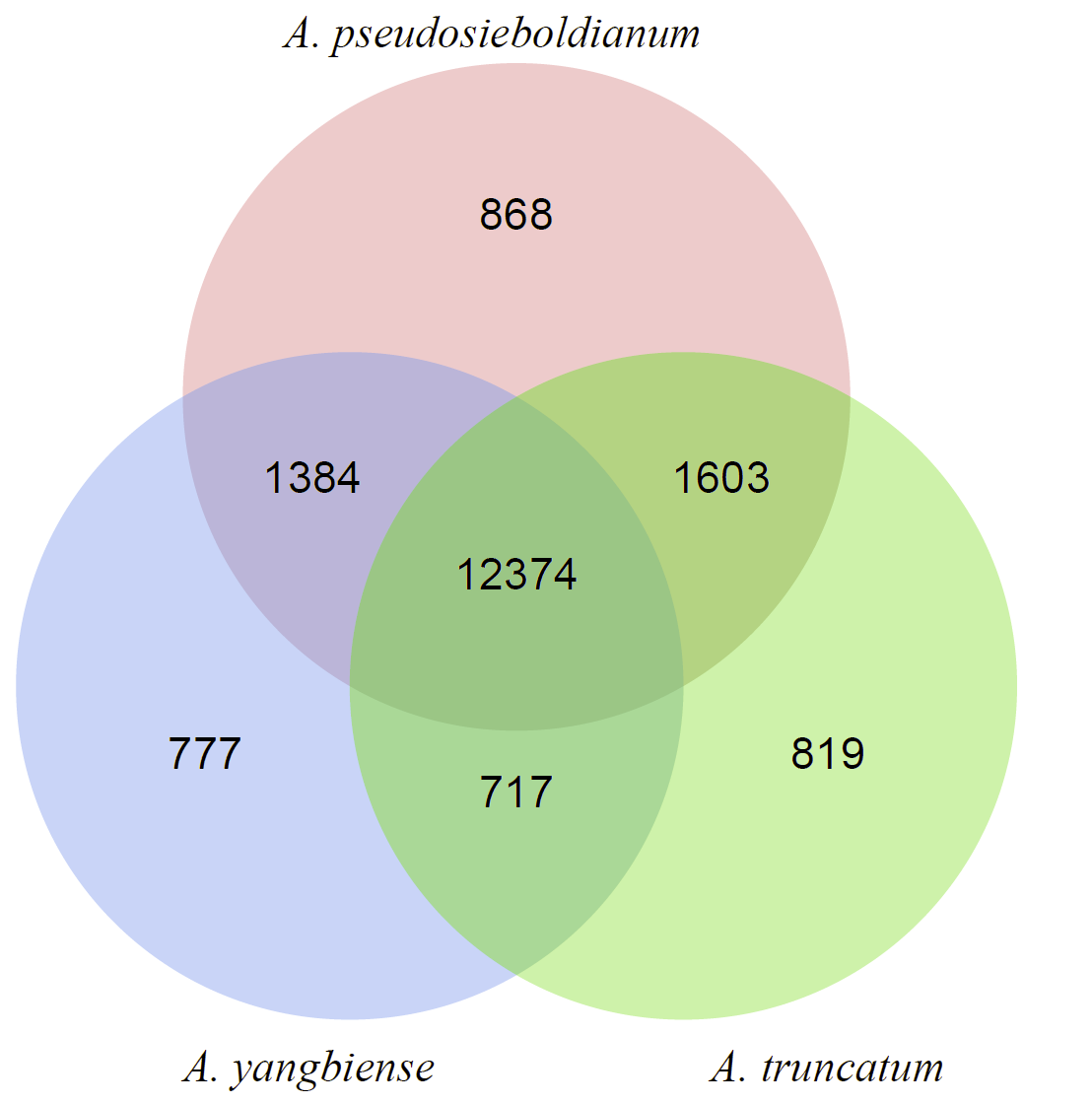


**Figure S5.** Venn diagrams of differentially expression gene family in *A. pseudosieboldianum*, *A. yangbiense* and *A. truncatum.*


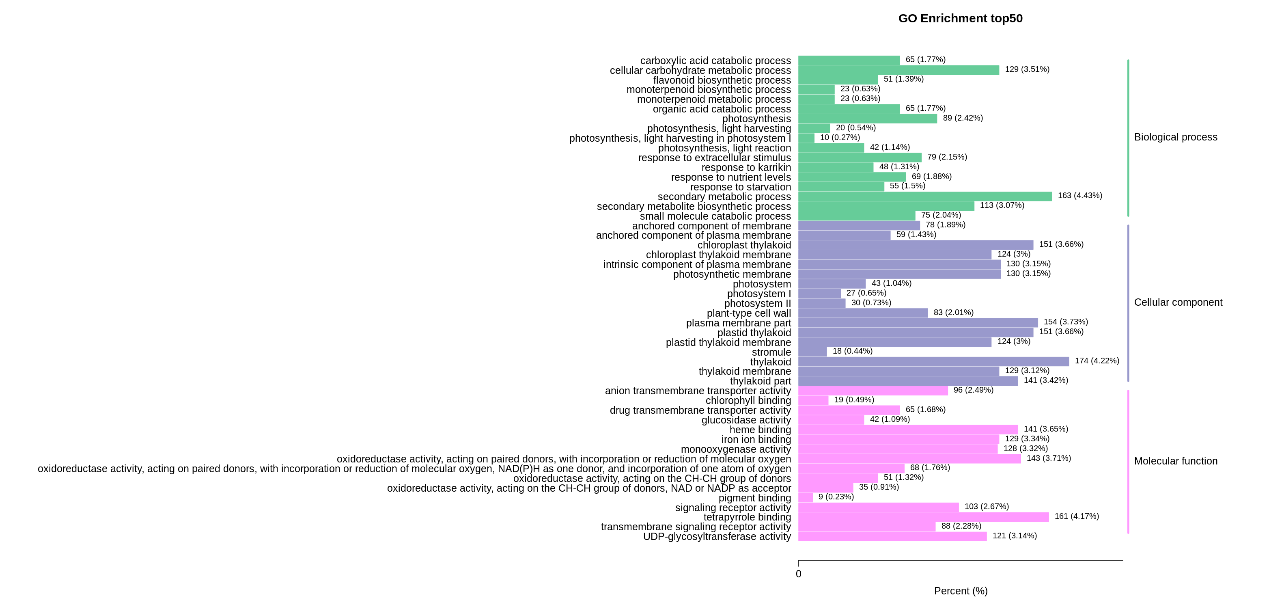


**Figure S6.** The GO enrichment analysis of GL vs RL during developmental fruit in *A. pseudosieboldianum*.


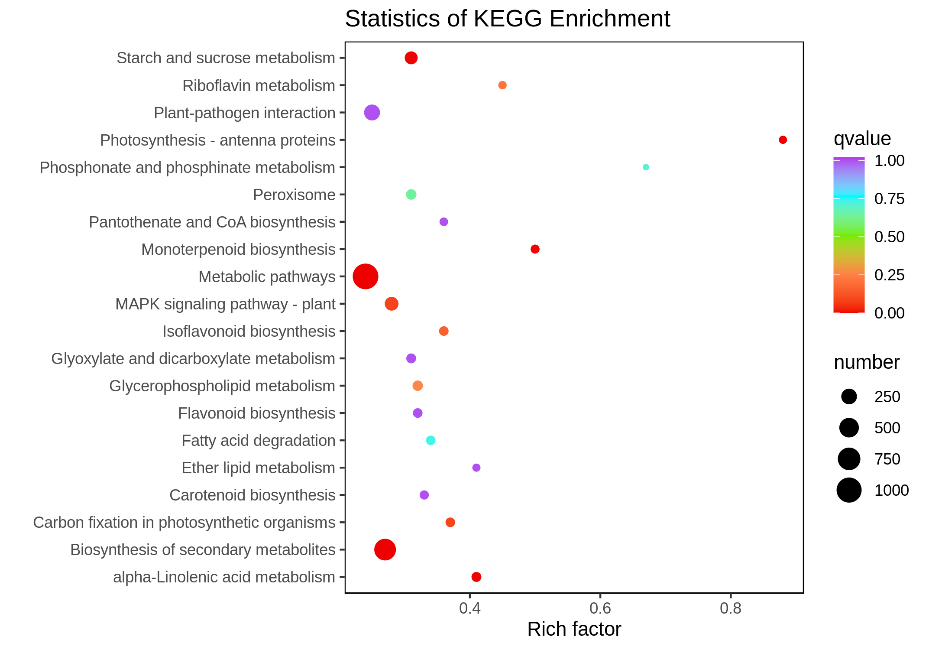


**Figure S7.** The KEGG enrichment analysis of GL vs RL during developmental fruit in *A. pseudosieboldianum.*


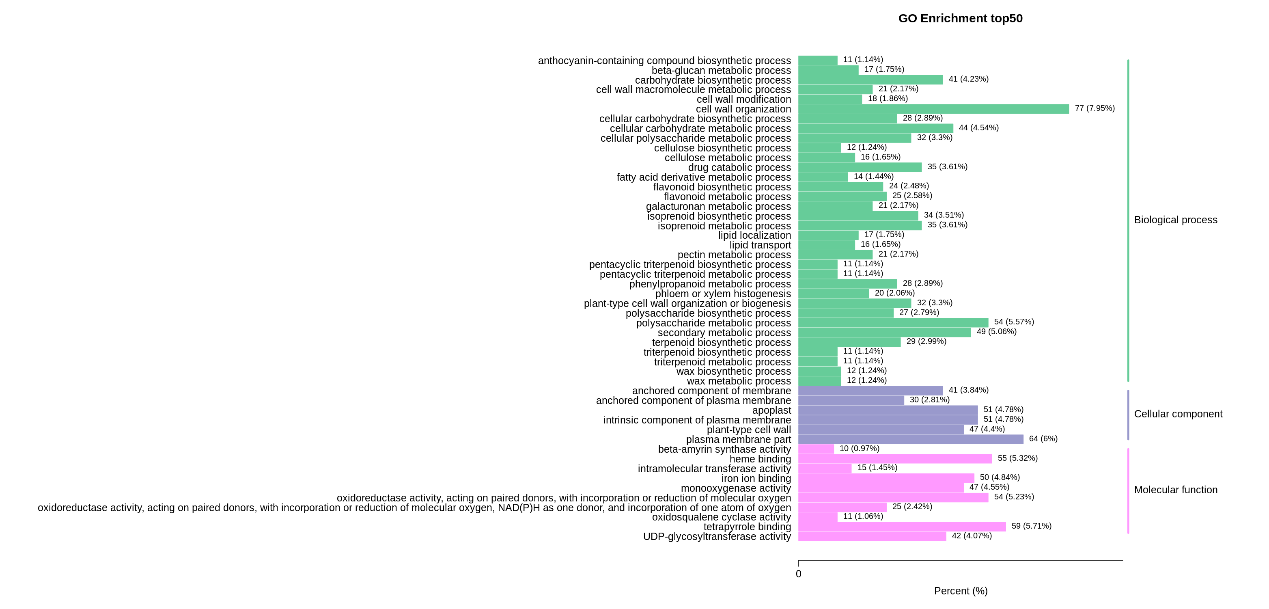


**Figure S8.** The GO enrichment analysis of GL vs HRL during developmental fruit in *A. pseudosieboldianum.*


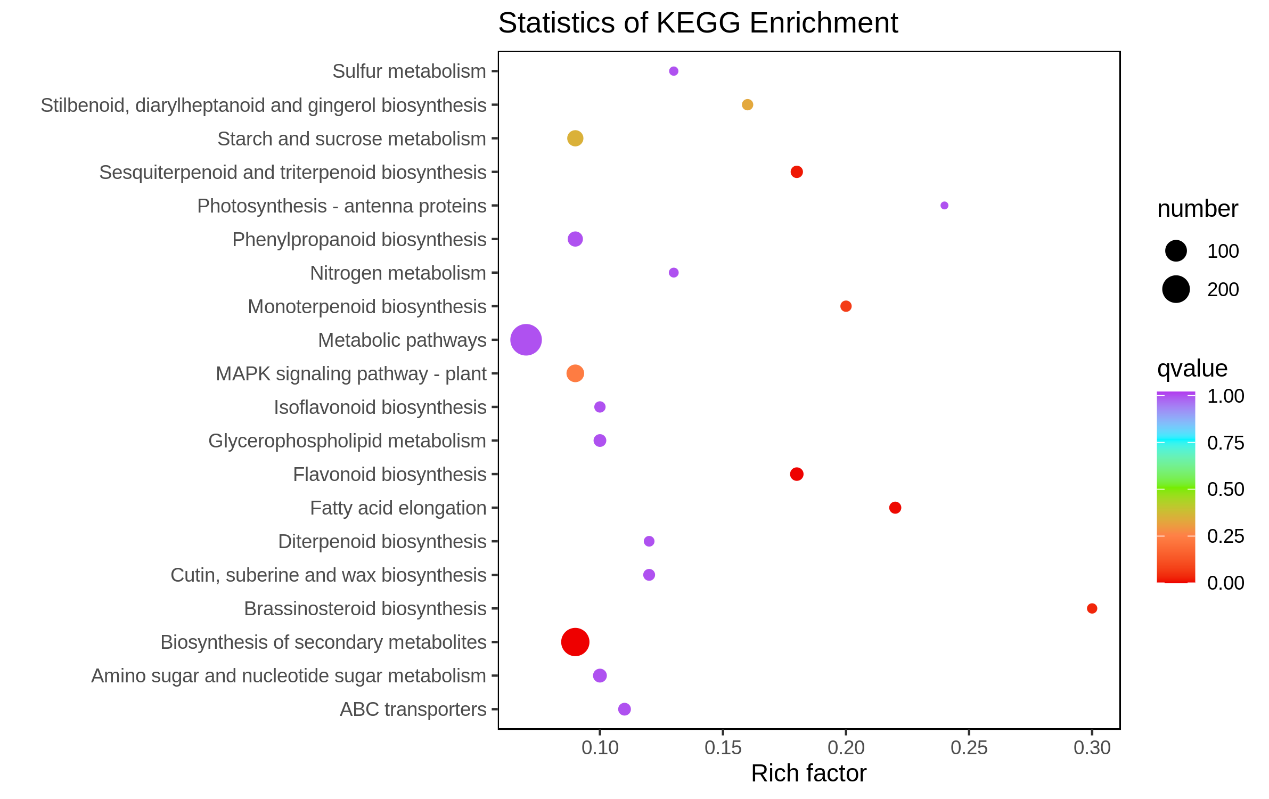


**Figure S9.** The KEGG enrichment analysis of GL vs HRL during developmental fruit in *A. pseudosieboldianum*.


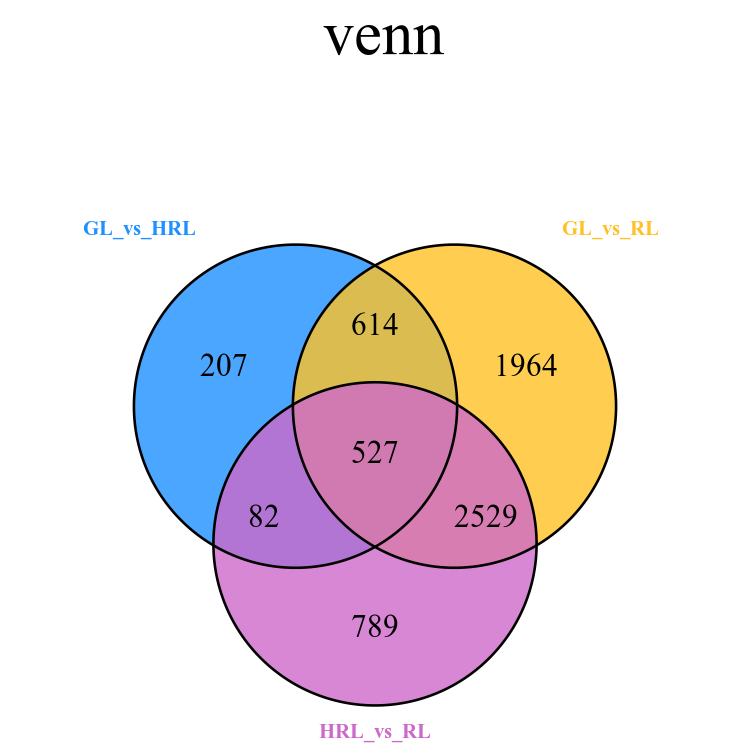


**Figure S10.** Venn diagrams of differentially expression genes in GL vs HRL, GL vs RL and HRL and RL


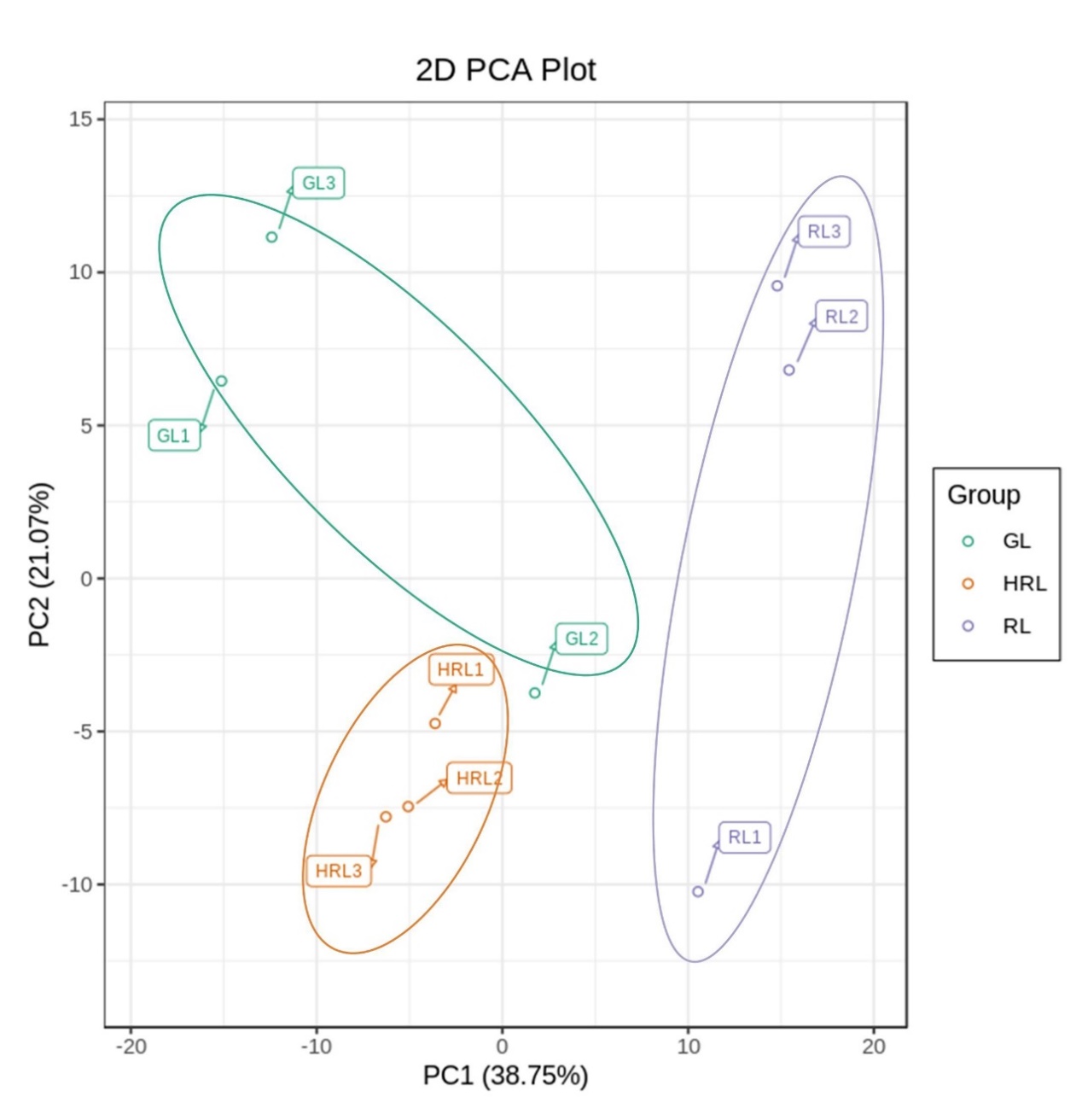


**Figure S11** PCA score plot metabolite profiles from different sample groups
